# Supramolecular Networks from Block Copolymers Based on Styrene and Isoprene Using Hydrogen Bonding Motifs—Part 1: Synthesis and Characterization

**DOI:** 10.3390/ma11091608

**Published:** 2018-09-04

**Authors:** Elaine Rahmstorf, Volker Abetz

**Affiliations:** 1 Institute of Physical Chemistry, University of Hamburg, Grindelallee 117, 20146 Hamburg, Germany; elaine.rahmstorf@chemie.uni-hamburg.de; 2 Institute of Polymer Research, Helmholtz-Zentrum Geesthacht, Max-Planck-Straße 1, 21502 Geesthacht, Germany

**Keywords:** anionic polymerization, triblock copolymer, modification, self-complementary hydrogen bonding, supramolecular polymers, amphiphilic polymers, temperature responsiveness

## Abstract

The combination of controlled anionic polymerization and subsequent introduction of hydrogen bonding groups was established to form thermo-reversible, supramolecular networks. Several polyisoprene-*block*-polystyrene-*block*-polyisoprene (ISI) copolymers—with polystyrene (PS) as the main block, and consequently giving the decisive material characteristics—were synthesized. The novel modification approach to post-functionalize the polyisoprene (PI) end-blocks and to introduce different motifs, which are able to form self-complementary hydrogen bonds, was attained. In the first step, hydroxylation was accomplished using 9-borabicyclo[3.3.1]nonane. Starting from the hydroxylated polymer, esterification with succinic anhydride was implemented to form an ester group with carboxylic end-group (-O-CO-CH_2_-CH_2_-COOH). In a second approach, 1,1’-carbonyldiimidazole was used as coupling agent to introduce various types of diamines (diethylenetriamine, triethylentetramine, and 2,6-diaminopyridine) to prepare urethane groups with amine end-group (-O-CO-NH-R-NH_2_). ^1^H NMR spectroscopy was used to confirm the successful synthesis and to calculate the degree of functionalization *D_f_*. Differential scanning calorimetry (DSC) showed a difference of the glass transition temperature *T_g_* between unfunctionalized and functionalized block copolymers, but no greater influence between the different types of modification, and thus, on the *T_g_* of the PS block. In temperature dependent FTIR spectroscopy, reversible processes were observed.

## 1. Introduction

Supramolecular polymers are formed by the assembly of lower-molecular-weight building blocks to macromolecules via non-covalent interactions. For their formation different non-covalent interactions can be used. Apart from ligand-metal complexation or π–π-stacking, especially hydrogen bonding is a promising approach [1,2,3]. An important characteristic of supramolecular polymers is their reversibility. Depending on the used modification, a responsiveness towards additives, pH value or temperature is created and a dynamic system is formed.

Most of the investigated supramolecular polymers consist of lower-molecular-weight building blocks or of statistically functionalized polymers with higher molecular weights [4,5]. The position of the interacting functional group within the polymer influences whether the polymer forms architectures like rods, lamellae, chains or networks, for example. While the α,ω-functionalization forms linear structures, statistically modified polymers build cross-linked networks. The group of Meijer [6] demonstrated that low molecular weight α,ω-end-group functionalized assemblies show polymeric, viscoelastic behavior.

Arrangements of several hydrogen bonding groups in one introduced side group are also called motifs. Their directional and highly specific character leads to defined complementary groups. The stability of the formed structures can be tuned over a wide range from weak to strong with interaction strengths ranging from a few to tens of kJ mol^−1^ [1,7]. Depending on the used type of functional groups, their arrangement, and the number of groups, well-defined polymers, and thus, characteristics from elastomeric over thermoplastic to highly cross-linked, can be achieved. In addition, depending on the functional groups of the motif, polymers with tunable temperature dependent properties can be obtained [1]. 

**Figure 1:**
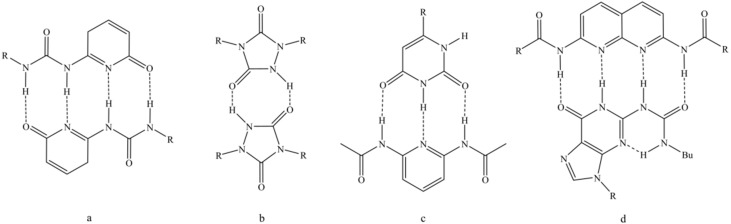
Well investigated examples for different multiple hydrogen bonding motifs. Self-complementary groups as (**a**) 2-ureido-4[1H]-pyrimidinone group (UPy) [10], (**b**) motifs with urazole units [13], (**c**,**d**) non-self-complementary groups with uracil and 2,6-diacyl-amino-pyridin type motifs [17] and deazapterin combined with UPy [18], respectively.

An important factor for the stability of these hydrogen bonded networks is the complex formation constant. It is influenced by the introduced hydrogen donor and acceptor groups as well as their spatial arrangement. The latter one is also considered by the secondary interaction parameter [8,9]. Multiple hydrogen bonding groups can be divided into self-complementary and non-self-complementary groups, which opens even more possibilities to get tunable mechanical properties. A strong quadruple-hydrogen-bonding dimer is the 2-ureido-4[1H]-pyrimidinone group (UPy) (Figure 1a), first used by Meijer’s group [10], which showed highly thermally responsive behavior [11,12]. Stadler’s group [13,14,15,16] used triazolindione chemistry to synthesize, for example, motifs with urazole units (Figure 1b), whereas Lehn’s group [17] worked with uracil and 2,6-diacyl-amino-pyridin type motifs (Figure 1c). Zimmermann’s group [18] combined deazapterin with UPy units (Figure 1d).

Not only non-covalent interactions, but also covalent bonding has to be mentioned [19]. The approach of using reversible, covalently bonded networks is implemented by the use of covalent adaptable networks (CAN), which can be divided into dissociative and associative CANs (based on the type of exchange reaction) [20]. The latter one includes the vitrimers, where several studies can be found in literature [21,22], especially by the groups of Leibler and Du Prez [20,23,24]. At room temperature, covalently bonded, high-molecular-weight networks consisting of lower-molecular-weight building units are present. At higher temperatures, dissolution of the network takes place, and thus, fluid like behavior dominates, comparable to that of a viscoelastic material. Re-cooling results again in network formation. As an example to modify polymers with dissociative CANs, one well investigated system is the (retro-) Diels-Alder reaction [25,26,27].

In this work, the idea of temperature-dependent and reversible mechanical behavior is transferred to block copolymers, modified with hydrogen bonding motifs. Consequently, characteristics of polymers with a much higher molecular weight than the original, unmodified polymer should be obtained at room temperature, and heating should lead to the dissolution of these networks. These reversible structures—with good processabilities—could be used, for example, as matrix in nanoparticle/polymer-composites to form hierarchical structures. Originating from the idea of Bayer and Stadler [28]—where end-blocks of polybutadiene-*block*-polystyrene-*block*-polybutadiene (BSB) triblock copolymers were modified by grafting on PEO segments—the question is how the complete functionalization of end-blocks is accomplished, and to what extent it is influencing the polymers characteristics. Thus, in this new approach, end-block functionalized polymers will be used to form supramolecular structures. The complete route from synthesis to analytics will be presented. Triblock copolymers, with a main PS middle block (>90 wt %), and small PI end-blocks—consisting of a few to several tens of monomeric units—will be used as starting material. Therefore, the starting material is synthesized via anionic polymerization, and thus, well defined polymers—in terms of molecular weight and composition—are obtained. In the second step, several functionalization reactions are performed on tri- as well as on diblock copolymers from styrene and isoprene. Depending on the polarity of the introduced groups and their number, the polymers become amphiphilic. The first modification is the hydroxylation, followed by esterification or amination to introduce carboxylic or amine functionalities. Therefore, established synthetic procedures from literature [29,30,31,32] had to be modified.

## 2. Materials and Methods

### 2.1. Materials

Block copolymers were synthesized from styrene (≥99% with 4-*tert*-butylcatechol as stabilizer, Sigma-Aldrich, Schnelldorf, Germany) and isoprene (99% with <1000 ppm 4-*tert*-butylcatechol as inhibitor, Sigma-Aldrich) in tetrahydrofuran (THF, >99%, with 3,5-di-*tert*-butyl-4-hydroxytoluene as stabilizer, VWR chemicals) using *sec*-butyllithium (*sec*-BuLi, 1.4 M in cyclohexane, Sigma-Aldrich) as initiator. Termination was accomplished with methanol (MeOH, 99.9%, Acros, Fairlawn, NJ, USA). As purification reagents *sec*-butyllithium (*sec*-BuLi, 1.4 M in cyclohexane, Sigma-Aldrich), *n*-butyllithium (*n*-BuLi, 1.6 M in hexane, Sigma-Aldrich) and di-*n*-butylmagnesium (Bu_2_Mg, 1.0 M in heptane, Sigma-Aldrich) were used. 

For functionalization reactions THF as well as 1,4-dioxane (DOX, 99%, with 3,5-di-*tert*-butyl-4-hydroxytoluene as stabilizer, Burdick und Jackson, Muskegon, MI, United States of America), 9-borabicyclo[3.3.1]nonane (9-BBN, 0.5 M in THF, Sigma-Aldrich), sodium hydroxide (NaOH, 100%, Grüssing, Filsum, Germany), hydrogen peroxide (H_2_O_2_, 30%, Merck, Darmstadt, Germany), succinic anhydride (SA, 99%, Sigma-Aldrich), 4-dimethylaminopyridine (DMAP, 99%, Sigma-Aldrich), 1,1’-carbonyldiimidazole (CDI, 100%, Sigma-Aldrich), diehtylentriamine (DETA, 100%, Sigma-Aldrich), triehtylentetramine (TETA, 100%, Sigma-Aldrich), and 2,6-diaminopyridine (DAP, 100%, Alfa Aesar, Haverhill, MA, USA) were used as received.

### 2.2. Anionic Polymerization

**Figure 2:**
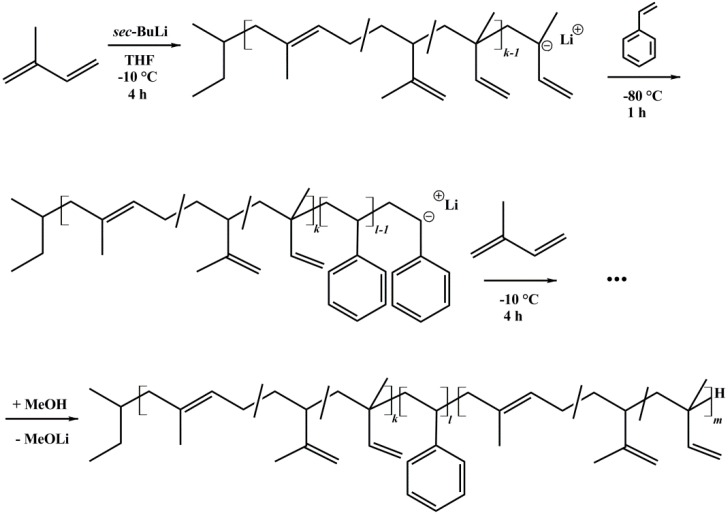
Reaction scheme of ISI triblock copolymers, synthesized via anionic polymerization in THF using *sec*-butyllithium as initiator.

Several asymmetric polyisoprene-*block*-polystyrene-*block*-polyisoprene (ISI) triblock copolymers and polystyrene-*block*-polyisoprene (SI) diblock copolymers were synthesized by sequential anionic polymerization in THF [33]. All syntheses were initiated with *sec*-BuLi. Before polymerization, THF was distilled and purified via titration with *sec*-BuLi [34,35]. Styrene was purified over Bu_2_Mg and polymerized at −80 °C for 1 h. Isoprene was purified two times over *n*-BuLi, and polymerized at −10 °C for 4 h. Termination was done with MeOH. All solvents and monomers were degassed prior to use. The polymer was precipitated in cold methanol. Notation of block copolymers is written as I*_x_*S*_y_*I*_z_*^M^, where *x*, *y*, and *z* correspond to the weight percent of the polystyrene and polyisoprene block, respectively. *M* indicates the number average molecular weight *M*_n_ in kg/mol [36]. The reaction scheme is shown in Figure 2.

### 2.3. End-Block Modification of Block Copolymers

In this work, the double bonds of polyisoprene were modified. Several functionalization reactions were carried out to obtain different kinds of hydrogen bonds.

#### 2.3.1. Hydroxylation

**Figure 3:**
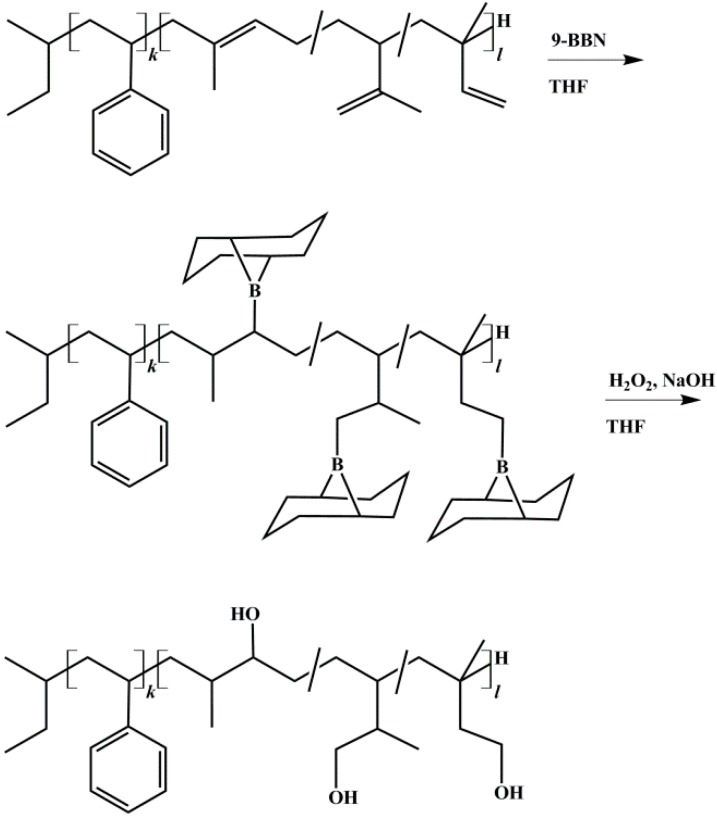
Reaction scheme for hydroxylation of block copolymers with 9-BBN (9-borabicyclo[3.3.1]nonane), shown for polystyrene-*block*-polyisoprene (SI) diblock copolymer. Anti-Markovnikov product is obtained due to large steric requirements of the alkyl borane during addition [40].

Hydroxylation was achieved according to literature using 9-BBN [29,37]. Polymers were vacuum dried several days at room temperature. THF was stored 48 h over molecular sieve 4 Å. All reaction steps were carried out under Schlenk conditions under nitrogen flow. Polymers were dissolved as 5 wt % solution and cooled to −20 °C. To 1 Eq. of vinyl and methylvinyl groups from the PI block the appropriate amount of 9-BBN was transferred into the flask. After 2 h the temperature of the solution was slowly raised to room temperature and stirred overnight. Then it was cooled to −30 °C before 5 Eq. (referring to 9-BBN) of anhydrous methanol were injected into the solution to deactivate excess of 9-BBN. After the solution was stirred for 30 min, a sample was taken and 1.05 Eq. (referring to 9-BBN) of 6 M NaOH were added. After additional 30 min, 1.5 Eq. (referring to NaOH) of 30% H_2_O_2_ were added. Now the solution turned opaque and partial precipitation of a white solid (NaOOH) occurred. After additional 2 h the temperature of the solution was slowly raised to room temperature and then to 55 °C. The solution was stirred for 1 h and turned clear. After cooling to room temperature, the polymer was precipitated in a mixture of methanol and water. During the oxidation step, the *B*-methoxy-9-BBN byproduct was oxidized to water soluble *cis*-1,5-cyclopentanediol and boric acid [38]. The latter forms a complex with methanol, and thus can also be removed by filtration. Its removal by distillation [39] (boiling point at 58 °C) showed no satisfying results in this work. The polymer was re-dissolved in THF, precipitated at least three times, and it was then vacuum dried at room temperature for several days. The reaction scheme of the hydroxylation process is shown in Figure 3. To exclude possible side reactions of PI double bonds during the oxidation process, reaction conditions of oxidation (see above) were applied to non-hydroborated, pure SI.

#### 2.3.2. Carboxylation

**Figure 4:**
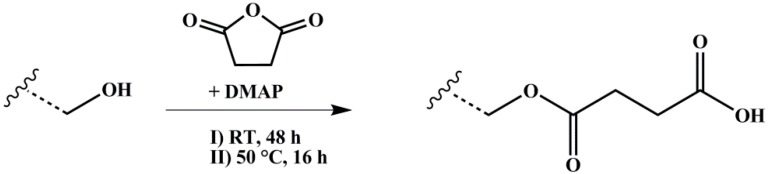
Reaction scheme for carboxylation of hydroxylated polymers with SA (succinic anhydride) and DMAP (4-(dimethylamino)pyridine) as catalyst. Wavy lines represent the polymeric main chain.

The polymer amount with 1 Eq. of hydroxylated PI units was solved with 10 Eq. of SA in dry THF and degassed several times. 2 Eq. of nucleophilic Steglich–Höfle catalyst DMAP were added and the reaction stirred for 6 h at 65 °C. After precipitation three times in methanol, the polymer was dried under vacuum at room temperature. The reaction scheme is shown in Figure 4.

#### 2.3.3. Amination

**Figure 5:**
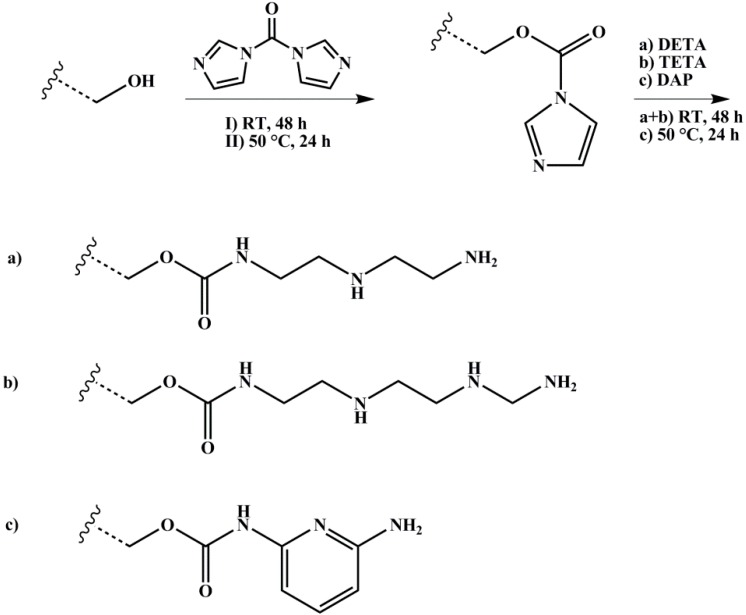
Reaction scheme for amination of hydroxylated polymers with different amines (DETA: diethylenetriamine, TETA: triethylenetetramine, DAP: 2,6-diaminopyridine) with 1,1’-carbonyldiimidazole (CDI) as coupling agent. Wavy lines represent the polymeric main chain.

The polymer with 1 Eq. of hydroxylated PI units was dissolved with 2 Eq. of CDI in dry DOX, degassed several times, and stirred over night at room temperature. 10 Eq. of DETA or TETA, respectively, were added and the reaction took place for four days at room temperature. In case of DAP, 10 Eq. were added, and the reaction ran for up to four days at 100 °C. After precipitation in methanol for three times, the polymer was dried under vacuum at room temperature. The reaction scheme is shown in Figure 5.

### 2.4. Characterization

#### 2.4.1. Size Exclusion Chromatography

The number average molecular weight *M*_n_ of the copolymers precursors from anionic polymerization as well as the dispersity index (*Đ*) of all functionalized polymers were determined by size exclusion chromatography (SEC). Narrowly distributed PS was used as standard. All SEC measurements were performed at 30 °C in THF on a PSS Agilent 1260 Infinity system equipped with a PSS SECcurity auto injector, and a PSS SECcurity isocratic pump with a flow rate of 1 mL/min (PSS Polymer Standards Service GmbH, Mainz, Germany). One pre-column and three analytical columns with porosities of 10^3^, 10^5^, and 10^6^ Å—consisting of modified styrene-divinylbenzene-copolymer gel columns as stationary phase (PSS Polymer Standards Service GmbH, Mainz, Germany)—were used. As internal standard, toluene was added. Detection of toluene was performed with UV-Vis wavelength detector at 260 nm (PSS SECcurity, light source: deuterium lamp, wavelength range of 190−600 nm). For polymer samples with concentrations of 1 mg/mL and an injection volume of 100 μL, a refractive index detector (PSS SECcurity differential-refractometer-detector) was used. Data processing was done with WinGPC UniChrom (PSS Polymer Standards Service GmbH, Mainz, Germany).

#### 2.4.2. Nuclear Magnetic Resonance Spectroscopy

Proton nuclear magnetic resonance (^1^H NMR) spectra of all polymers were recorded on a Bruker AVANCEII (Bruker BioSpin GmbH, Karlsruhe, Germany) at 400 MHz. Tetramethylsilane (TMS) was used as internal standard, and chloroform-d1 (CDCl_3_) or tetrahydrofuran-d8 (THF-d8) as solvent. Sample concentration was 10–20 mg/mL. Measurements were recorded at 300 K. Data processing was carried out with MestReNova (Version 7.1.0, Mestrelab Research S.L., Santiago de Compostela, Spain).

#### 2.4.3. Fourier Transform Infrared Spectroscopy

FTIR experiments were recorded on a Bruker FTIR Vertex 70. Measuring software was Opus 7.5. All samples were measured in the wavenumber range of 6000–400 cm^−1^ with a resolution of 2 cm^−1^ and 32 scans. For temperature dependent FTIR experiments, samples were prepared via solution casting of a polymer film (ca. 1–2 mg in THF) coated onto a 6 mm by 1 mm potassium bromide plate. Films were dried for 16 h under vacuum at room temperature. The temperature profile was chosen as follows: First heating cycle from 30 to 110 to 30 °C, and then, second heating cycle, from 30 to 190 to 30 °C. Temperature steps were 10 °C with a 15 min isothermal hold between successive steps in the temperature cycle to ensure equilibrium. A background measurement without specimen in the sample holder was carried out at 30 °C and subtracted from the recorded data by the Bruker software OPUS. No additional data processing was implemented. 

#### 2.4.4. Differential Scanning Calorimetry

To determine the glass transition temperature *T_g_* of unfunctionalized and functionalized block copolymers, a differential scanning calorimeter DSC 1 (Mettler-Toledo, Greifensee, Switzerland) and a DSC 204 F1 Phoenix (NETZSCH-Gerätebau GmbH, Selb, Germany) were used. Therefore, 5–10 mg polymer was weighed into an aluminum crucible, slightly pressed and then closed afterwards. The measurements were performed at 1 bar under nitrogen atmosphere (flow rate of 20 mL/min) in the temperature range between −150 °C and 200 °C. The heating and cooling rates were 5 °C/min. During a first heating interval the thermal history of block copolymers was erased by heating up the samples from room temperature to 150 °C, followed by cooling them down to −150 °C. In the second interval they were heated to 150 °C. After cooling down to −150 °C, in the third interval they were heated to 200 °C. The thermal properties were analyzed using the DSC data of the second and third heating. Data processing was performed by STARe software (Mettler-Toledo, Version 12.10a), and by Proteus analysis (NETZSCH-Gerätebau GmbH, Selb, Germany).

#### 2.4.5. Thermogravimetric Analysis

A TGA 209 F1 Iris (NETZSCH-Gerätebau GmbH, Selb, Germany) was used to determine the mass loss during heat treatment. Temperature range of 25–300 °C with a heating range of 5 °C/min was chosen. About 5 mg of polymer were weighed into a ceramic crucible. Dynamic nitrogen atmosphere was used with a flow of 20 mL/min. Data processing was performed with NETZSCH Proteus-Software.

#### 2.4.6. Small Angle X-ray Scattering

SAXS measurements were performed to determine the phase separation of the block copolymers. Therefore, films of about 1 mm thickness were prepared by solvent annealing (of THF solutions)—with and without further heat treatment above *T_g_*—as well as by melt pressing (specimen was heated without vacuum for 3 min, under vacuum for 1 min, and finally, under vacuum and an applied force of approximately 55 kN for 4 min). An Incoatec X-ray source IμS with Quazar Montel optics, a focal spot diameter at the sample of 700 mm with a wavelength of 0.154 nm, and a Rayonix SX165 CCD-Detector were used for these measurements. The distance between the sample and detector was 1.6 m.

## 3. Results and Discussion

In this work, results from different diblock (SI) and triblock (ISI) copolymers with molecular weights from 50–150 Da and overall PI weight fractions up to 59 wt %—with a main focus on the range from 1.5–15 wt %—are presented. Table 1 shows the determined compositions, degree of polymerization *P*_n_ and dispersity indices *Đ*. ^1^H NMR spectra of precursor and final product are given in the supporting information (Figure S1).

The nomenclature in this work is done as follows: The main block copolymers are labelled as described earlier, for example I_5_S_90_I_5_^62^. To ease comparability, polymer compositions are not recalculated after modification. Introduced functional groups are indicated with a suffix at the original polymer. The suffix -OH defines the hydroxylated block copolymer. ISI-DETA, ISI-TETA, and ISI-DAP, respectively, define the different kinds of aminated polymers (diehtylenetriamine, triehtylenetetramine, 2,6-diaminopyridine). With succinic anhydride (SA) carboxylated ISI is labelled as ISI-SA. The degree of functionalization *D_f_* defines the ratio of functionalized PI monomer units in comparison to the unfunctionalized PI. *D_f_* is not related to the PS block.

### 3.1. Synthesis of Modified Block Copolymers and the Calculation of Degree of Functionalization D_f_

The reactions were chosen according to an efficient and a preferably easy preparation. To introduce hydroxyl groups, the use of 9-BBN as functionalization reagent seemed to be a good choice. For example, in comparison to epoxidation/hydrolysis [41] experiments, higher conversions were achieved. With the use of anhydrides for Steglich esterification, and due to higher reactivity of the anhydride compared to the acid form, no DCC (*N*,*N*’-dicyclohexylcarbodiimide) is needed to increase the reactivity of this component, and thus no dicyclohexylurea is formed during the reaction, which would have to be removed. Consequently only DMAP was used. For the introduction of amine-containing motifs, the use of CDI as coupling agent seemed to be very promising [42]. Unfortunately, not all used amines showed the same good conversions with the CDI-activated polymers.

**Figure 6:**
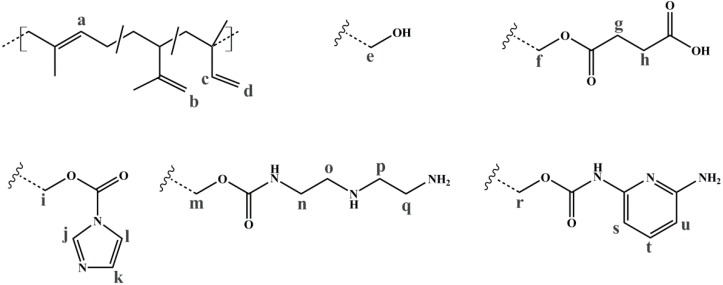
Used indices of the ^1^H NMR related protons, corresponding to each motif. The indices are directly inserted into the ^1^H NMR spectra in Figure 7 and Figure 8.

**Figure 7:**
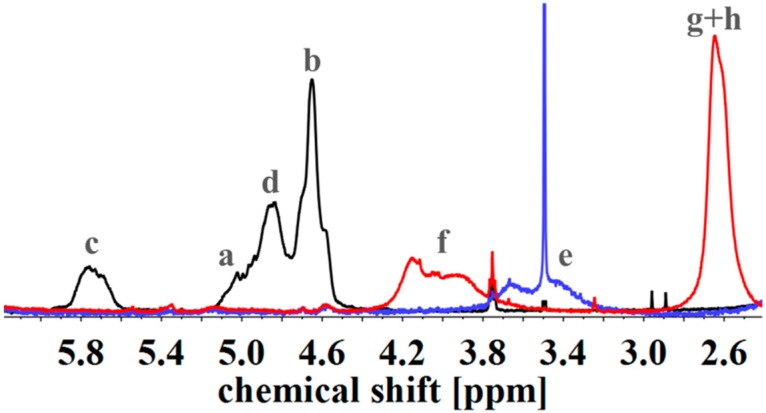
^1^H NMR spectra of unfunctionalized (black), hydroxylated (blue) and with SA carboxylated (red) I_5_S_90_I_5_^62^ in CDCl_3_. Spectra were normalized to aromatic protons of PS (6.2–7.2 ppm, 5H).

**Figure 8:**
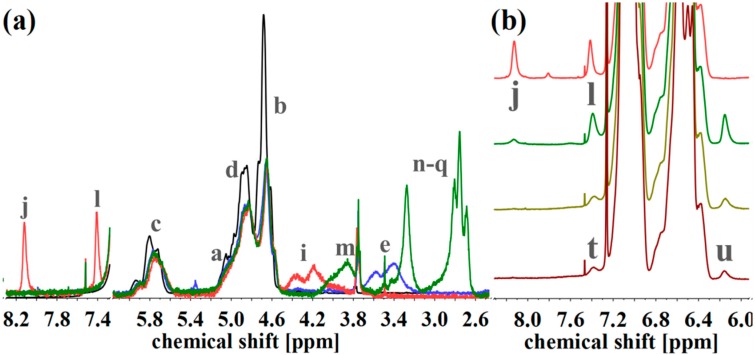
(**a**) ^1^H NMR spectra of S_85_I_15_^51^ (black), after hydroxylation (blue), and after reaction with CDI (orange) and DETA (green) in CDCl_3_. (**b**) ^1^H NMR spectra of CDI-functionalized S_85_I_15_^51^ (top), and after addition of DAP with reaction times of 7 h, 3 d and 4 d (from top to the bottom) in CDCl_3_. ^1^H NMR spectra were normalized to aromatic protons of PS (6.2–7.2 ppm, 5H).

To evaluate the successful introduction of different functional groups, and to determine the *D_f_*, ^1^H NMR spectroscopy was used as an analytical tool. For all functional groups specific signals can be observed. To facilitate the signal assignment, Figure 6 shows the characteristic protons of each motif, including the PI microstructures. The proton related indices are directly inserted into the ^1^H NMR spectra of Figure 7 and Figure 8. According to its two double bonds, isoprene can form different configurations after polymerization. In THF as polymerization solvent, 1,2-, 3,4-, and *trans*-1,4-PI are obtained [43,44]. For each microstructure a different chemical shift can be observed. The signal “c” at ~5.8 ppm can be assigned to –C**H**=CH_2_, and ~4.8 ppm to the two protons “d” of –CH=C**H**_2_ (both 1,2-PI). The two protons “b” of –C(CH_3_)=C**H**_2_ (3,4-PI) create the signal at 4.6 ppm. The proton “a” of –C**H**=C(CH_3_)-(*trans*-1,4-PI) can be observed at ~5.1 ppm [45,46].

To calculate *D_f_*, the following described approach was chosen. All spectra were normalized to the aromatic proton signal of PS (6.2–7.1 ppm). For hydroxylated polymers (Figure 7, for example), *D_f_* was calculated via the decrease of characteristic PI signals in the range of 4.4–6.1 ppm. ^1^H NMR spectra of unfunctionalized and hydroxylated polymer, considering the different microstructures, were compared. The characteristic signal between 3.2 and 3.8 ppm, arising from the protons “e” adjacent to the hydroxy group (–C**H***_x_*-OH), was not chosen [37,47]. Due to different microstructures of PI, this signal was generated by one or two protons, and thus, calculations were more complex and with a larger error than the first approach. In order to ensure that no side reactions influenced the decrease of double bonds, several test reactions were done. For example, after the addition of sodium hydroxide and hydrogen peroxide under hydroboration/oxidation reaction conditions (see experimental part), no obvious changes in ^1^H NMR spectra were observed. For the further functionalized polymers, *D_f_* was calculated with regard to specific signals. The degree of polymerization, and hence the number of PS monomer units, was set as a constant value. Thereupon, characteristic signals of the introduced motifs were evaluated with regard to the aromatic signal of PS. Because of the signal-to-noise-ratio of the spectra, and the possibility of less than 100% conversion, *D_f_* of amine- or carboxyl-functionalized polymers was always smaller than the *D_f_* of hydroxylated polymers. In general, for all further modifications a shift of the signal at 3.2–3.8 ppm was observed.

For SA-functionalized polymers, the signal at 2.4–2.8 ppm, related to the four protons “g + h” of the ethylene group [48] in the formed side chain, is used (Figure 7). In addition, the high field shift of the signal –C**H***_x_*-O-C=O– from 3.2–3.8 ppm (“e”) to 3.7–4.3 ppm (“f”) is apparent. For this block copolymer, no significant signals related to PI double bonds are left after hydroxylation.

For the amine-functionalized polymers—for example the S_85_I_15_^51^-DETA (Figure 8a)—the signal at 2.5–3.6 ppm, related to the eight protons of the ethylene group in the formed side chain, is used for the calculation of *D_f_*. Again, also the shift of the –CH*_x_*-O-C=O– proton signal from 3.2–3.8 ppm (“e”) to 4.0–4.5 ppm (“i”) (for SI-CDI), and to 3.6–4.2 ppm (“m”) (for SI-DETA) is apparent. In this example, PI double bonds are still present, indicating a degree of hydroxylation less than 100%. For the DAP-functionalized polymers (Figure 8b), two of three characteristic signals can be observed. Only one of them can be used for the calculation of *D_f_*. The proton closer to the carbamate shows a signal at 7.0 ppm and is overlaid by the aromatic PS signal. The proton “t” in *para*-position leads to the signal at 7.4 ppm, and—depending on conversion—is overlapping with the signal arising from the CDI-functionalized polymer (“l”). The aromatic proton closer to the terminal amine (“u”) is located at 6.2 ppm, and can be used for further calculations. Figure 8b shows the spectra of CDI-functionalized S_85_I_15_^51^ (top), and after addition of DAP with reaction times of 7 h, 3 days and 4 days (downwards).

All mentioned characteristic signals can be observed. 7 h after addition of DAP (Figure 8b, dark green), signals of CDI still can be seen, and signals of DAP show their maximum. After 3 days (Figure 8b, light green) the CDI related signal at 8.2 ppm is remaining negligibly. Unfortunately, too long reaction times showed a decrease of DAP-specific signals, thus the temperature-induced decomposition of the urethane motif took place. The *D_f_* for the S_85_I_15_^51^-DAP decreases from 18% (after 7 h), 12% (after 3 days), to 4% (after 4 days).

For the calculation of *D_f_* (in %) of the functionalized polymers, the sum of all PI microstructure integrals (per proton) *I*_*H,PI*_ were set as initial value. This value (representative for number of PI monomer units) was assumed to be constant. For all other functionalized polymers the integrals (per proton) *I*_*H,funct.*_ of characteristic signals were brought into relation (Equation (1). All calculated values are listed in Table 2.

(1) $$\;D_{f}=\frac{I_{H,funct.}}{I_{H,PI}}\cdot 100\%$$

**Figure 9:**
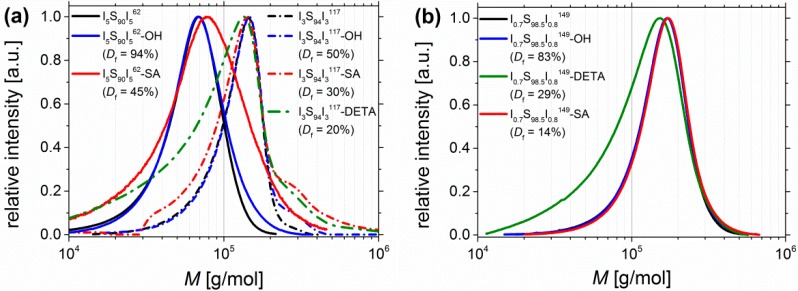
SEC curves of (**a**) I_5_S_90_I_5_^62^ (lined) and I_3_S_94_I_3_^117^ (dashed), and of (**b**) I_0.7_S_98.5_I_0.8_^149^ before functionalization (black), after hydroxylation (blue), after DETA-functionalization (green), and after carboxylation (red). I_5_S_90_I_5_^62^-DETA was not soluble at 30 °C, consequently no SEC was done. Calibration of SEC was done with polystyrene standards.

The obtained SEC curves of I_5_S_90_I_5_^62^, I_3_S_94_I_3_^117^ and I_0.6_S_98.5_I_0.7_^149^—before and after functionalization—are plotted in Figure 9. Related data are also listed in Table 2. For an easier comparison, the molecular weight distribution was preferred instead of the elution volume. It has to be taken into account that PS standards were used for calibration, and that the introduction of motifs leads to a change in the hydrodynamic radii, which influences the detected retention time—and consequently also the measured molecular weight distribution—of the investigated block copolymers.

For carboxylated polymers (Figure 9, red), only for high amounts of functional groups (I_5_S_90_I_5_^62^-SA) a shift towards higher molecular weights can be observed, which indicates the increase of the hydrodynamic volume after SA-functionalization. All other curves of functionalized I_5_S_90_I_5_^62^ remain similar. For block copolymers with 6–10 wt % PI, after carboxylation the molecular weight distribution is broadened—to lower *M* as well as to higher *M*. Interactions of column material and motif would increase the elution volume, and thus, decrease the detected molecular weight. In contrast, the formation of intermolecular hydrogen bonds would increase the detected molecular weight. However, only the DETA-functionalized polymers (Figure 9, green) show a larger shift towards smaller molecular weights, even for lower amounts of functional groups (I_0.7_S_98.5_I_0.8_^149^-DETA, Figure 9b, green). The “back-tailing” to lower *M*—thus higher elution volume—can also be attributed to interactions with the column material as well as to a reduced hydrodynamic volume. Both reasons lead to higher retention times. The shoulder of I_3_S_94_I_3_^117^-SA at 2*M* was also observed for its precursor I_3_S_94_I_3_^117^-OH, but less pronounced. The investigation of the hydroborated intermediate stage revealed the same shoulder. This observation leads to the assumption that—during hydroboration—partially chemical cross-linking (mostly coupling between two chains) occurred to a low extent. This possibility was already mentioned in the synthesis section. The 2*M* shoulder was not observed for other SA-functionalized ISI, and hence, should be not related to the carboxylation reaction. Except I_5_S_90_I_5_^62^-DETA, no polymers showed difficulties—as, for example, strong swelling after solvent addition—during the dissolution process, which could be an indication of strong physical or chemical cross-linking. Finally, an extreme broadening to high molecular weights (showing a multiple of *M*)—explained by chemical cross-linking—was not detected.

#### Adjustability of Degree of Functionalization *D_f_*

**Figure 10:**
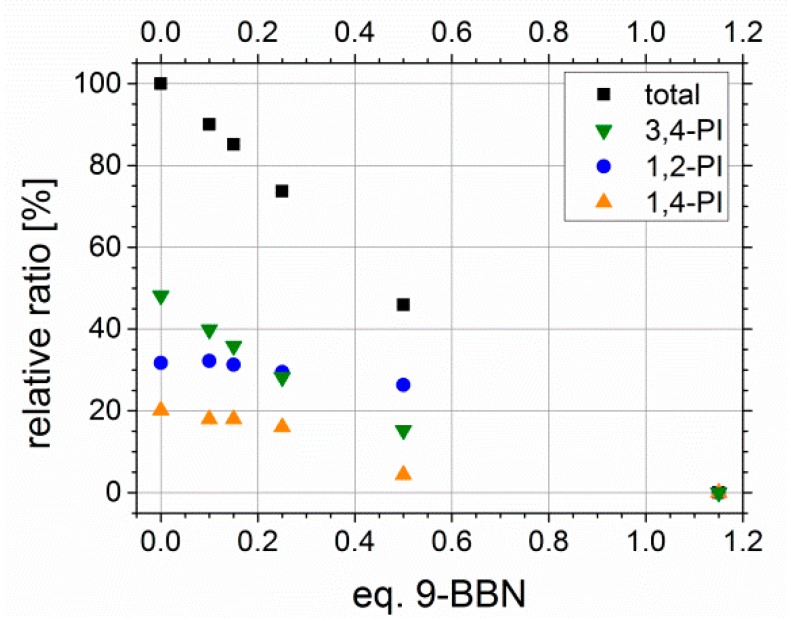
Conversion of polyisoprene double bonds in total, and divided according to microstructure (1,2-, 3,4- and *trans*-1,4-PI), depending on the used equivalents of 9-BBN. Used original polymers were S_41_I_59_^31^ and S_51_I_49_^5^.

Former hydroxylation experiments with different amounts of 9-BBN showed a linear behavior, and thus, the tunability of *D_f_* with varying amount of hydroboration agent. In Figure 10, the relative ratios of all PI microstructure double bonds in sum (black) as well as for each microstructure separately, depending on the used equivalents of 9-BBN, are presented. The equivalents of 9-BBN are quoted with regard to the used molar amount of PI double bonds. For higher contents of PI (about 50 wt %), the linear dependency known from literature [39] could be confirmed. The purification of the sample prepared with 0.8 Eq. 9-BBN revealed difficulties in finding an appropriate dissolution/precipitation agent to avoid micelle formation during this step, and thus, analysis was not feasible.

Unfortunately, this trend was not observed for block copolymers with lower PI contents. With less than 10 wt % the used equivalents of 9-BBN had to be increased. A *D_f_* of 100% never has been reached quantitatively, even with 5 Eq. of 9-BBN. One possible explanation is, that due to the low numbers of double bonds in comparison to styrene units, the sterical hindrance of neighboring phenyl rings from PS, and thus, the more difficult addition of sterically demanding 9-BBN has a greater impact on the resulting *D_f_*. Furthermore, this may is enhanced by a weaker segregation of PS and PI in solution due to the lower PI contents. Finally, this shows that double bonds still can exist inside the polymeric framework.

### 3.2. Thermal Characterization

**Figure 11:**
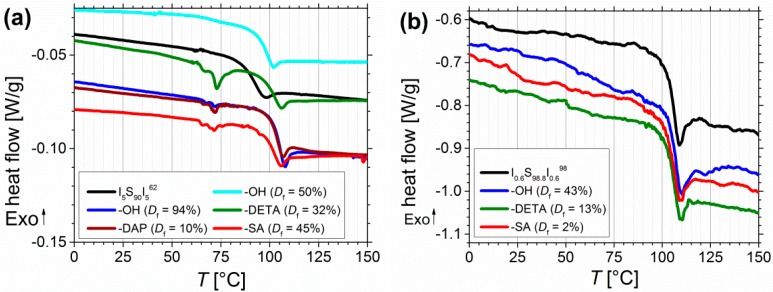

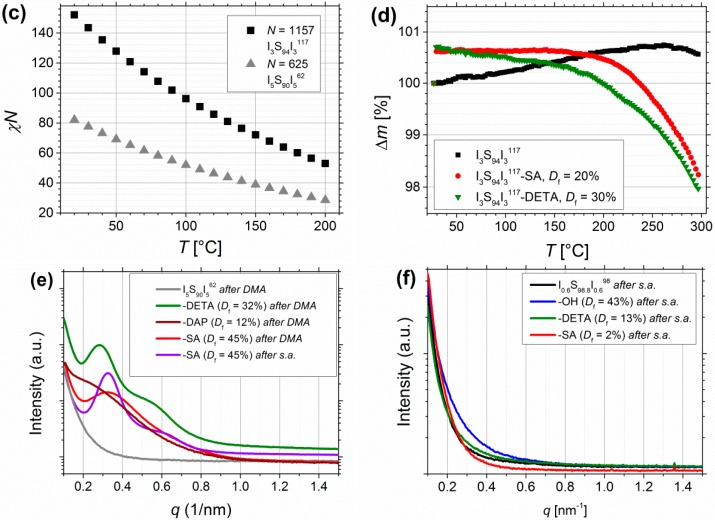
(**a**) DSC curves of pure I_5_S_90_I_5_^62^ (black), after functionalization with OH- (blue), DETA-(green), DAP-(brown) and SA-groups (red). Data were recorded on Mettler-Toledo DSC 1, and additionally processed for smoothening. (**b**) DSC curves of I_0.6_S_98.8_I_0.6_^98^ before and after functionalization with OH-(blue), DETA-(green), and SA-groups (red). Data were recorded on NETZSCH DSC 204 F1 Phoenix with no additionally data processing. (**c**) temperature-dependent, calculated values of *χN* for I_5_S_90_I_5_^62^ and I_3_S_94_I_3_^117^. (**d**) TGA of unfunctionalized I_3_S_94_I_3_^117^ (black, 5 mg), followed by carboxylated (*D_f_* = 30%, red, 7.2 mg), and DETA-functionalized ISI (*D_f_* = 20%, green, 4.6 mg). SAXS curves of I_5_S_90_I_5_^62^ (**e**) and I_0.6_S_98.2_I_0.6_^98^ (**e**) films before and after modification (*after DMA*: after angular frequency dependent oscillatory measurement at 120–180 °C, *after s.a.*: after solvent annealing).

DSC measurements were performed to determine the *T_g_*, and to investigate its shift due to different modifications. The onset of *T_g_* for different polymers before and after functionalization is also listed in Table 2. Figure 11 shows the data of the third DSC heating cycles of selected samples.

For all polymers only one *T_g_* was detectable. Before functionalization, for I_5_S_90_I_5_^62^ (Figure 11a) the onset of *T_g_* can be found at 86 °C. With increasing degree of hydroxylation *T_g_* shifts to 93 °C (*D_f_* = 50%) and to 101.5 °C (*D_f_* = 94%). For further modifications *T_g_* is observed at similar temperatures between 97 and 101 °C. One explanation can be found in the miscibility of the blocks. Before functionalization no phase separation of PS and PI blocks occurs, and thus, only one mixed *T_g,mix_* is detected. After functionalization, the *T_g_* of the pure PS block can be observed. To calculate the missing *T_g_* of the pure PI block, several approaches can be found in literature [49,50]. Equation (2) shows the Fox-Flory equation [51], which is commonly used for completely miscible polymers, with *ω* as the weight fraction of PI and PS, respectively. Temperature is given in Kelvin.

(2) $$\;\frac{1}{T_{g,mix}}=\frac{\omega_{PI}}{T_{g,PI}}+\frac{\omega_{PS}}{T_{g,PS}}$$

For example, before functionalization for I_0.7_S_98.5_I_0.8_^149^ a *T_g,mix_* of 99.6 °C was detected. After functionalization a *T_g_* of 102 and 103 °C was measured, which can be assigned to the pure PS block, and corresponds very good to data from literature [52,53]. After inserting both values into Equation (2), a *T_g,PI_* of –10 and –40 °C is calculated. For I_3_S_96_I_3_^117^, *T_g,PI_* values of –50 °C to –70 °C were obtained. Regarding the high content of 1,2- and 3,4-PI, and the low molecular weight of the PI block, these values fit well to data from literature. Depending on the molecular weight and the composition of the PI, –30 to –60 °C were detected [54,55]. In general, for lower PI contents (for example I_0.6_S_98.8_I_0.6_^98^) the shift of *T_g_* is comprehensible less pronounced than for I_5_S_90_I_5_^62^ (Figure 11a,b). The microphase separation between PS and the functionalized PI blocks could explain, why no greater influence of hydrogen bonds on the *T_g_* of PS was observed. In literature, for example, modified polybutadiene [56] showed an increase and broadening of *T_g_*—due to physical cross-linking—with increasing degree of functionalization, detectable up to 10% modification. In addition, Chung et al. [39] obtained a higher *T_g_* with increasing degree of hydroxylation, due to inter- and intramolecular hydrogen bonding. Concerning the low weight fraction of the functionalized blocks, and consequently the low number of functional groups, in this work, no further transitions from the PI blocks—neither before nor after functionalization—were detected in DSC. The observed endothermic transitions at 60–80 °C (Figure 11a) might be related to opening of hydrogen bonds, but they were only detectable on the Mettler-Toledo DSC 1. Appearance of artefacts should be excluded as these transitions occurred only for amine- and carboxyl-functionalized polymers. With a NETZSCH DSC 204 these transitions of modified I_5_S_90_I_5_^62^ were measureable, but with a smaller resolution, and thus, less pronounced. Finally, for all block copolymers no other transitions (for example the *T_g,PI_*, melting or decomposition peaks) could be observed.

Calculation of *χN*—where *χ* is the Flory–Huggins–Staverman interaction parameter of PS and PI, and *N* is the overall degree of polymerization—can be used to estimate the microphase separation behavior of block copolymers. Values were calculated by Equation (3) from Mori et al. [57]. Figure 11c shows the calculated values for I_5_S_90_I_5_^62^ and I_3_S_94_I_3_^117^ at different temperatures.

(3) $$\;\chi N=-0.0937+\;\frac{66}{T\left[K\right]}$$

With regard to the different phase separation behavior of symmetrical triblock copolymers in contrast to diblock copolymers, which was investigated by Matsen [58], it can be seen that the transition from disordered to ordered structures for symmetrical triblock copolymers is shifted to higher *χN* values. With the very low volume fractions of PI less than 10 vol%, the compositions of the current investigated triblock copolymers are positioned at the margin of the phase diagram. As presumed from DSC, microphase separation seems to occur in the first place after functionalization, where *χN* is probably significantly enhanced due to insertion of larger difference in polarity. This is confirmed by first SAXS studies on films prepared from solvent annealing as well as from melt pressing. Figure 11e confirms for I_5_S_90_I_5_^62^ that microphase separation is induced after introduction of different motifs—for films prepared via melt pressing (Figure 11e, red) as well as from solvent annealing (Figure 11e, purple). In contrast, for I_0.6_S_98.8_I_0.6_^98^ (Figure 11f) also after functionalization no microphase separation is observed, presumable due to too low contents of PI and motifs, respectively.

For further thermal characterization, thermogravimetric analysis (TGA) was performed (Figure 11d). A temperature range of 25–300 °C with a heating rate of 5 °C/min, and about 5 mg of sample were chosen. For unfunctionalized ISI no relevant change of mass is observed. For carboxylated samples, decomposition starts at around 150–160 °C, and continues until the end of measurement. For DETA-functionalized samples, decomposition starts also at around 160 °C and continues until the end of measurement. The mass loss increases at temperatures higher than 220 °C. Nevertheless, due to very low changes in mass (under 2%) because of the very low number of functional groups in contrast to the overall degree of polymerization, these measurements can be used only as an indication of material decomposition. For further measurements, larger amount of sample—to be inside the determination limit—and higher end temperatures are necessary.

### 3.3. Fourier Transform Infrared Spectroscopy

Infrared spectroscopy was used to validate the introduction of specific bands of different functional groups as well as to investigate the temperature dependent characteristics. All shown spectra are raw data, and except subtracting the background measurement, no additional data processing was applied. FTIR spectra of complete wavenumber range are available in the supplementary information (Figures S6 and S7).

Several characteristic bands can be used for the presented system. In this work, the main focus will be on the carbonyl region [59,60], where complexed and free species can be distinguished. Besides that, the typical bands of the different PI microstructures are located at 890 cm^−1^ and 1645 cm^−1^ for 3,4-PI, at 907.5 cm^−1^ for 1,2-PI, and at 840 cm^−1^ for 1,4-*trans*-PI [46,61,62]. These bands are overlapping with other signals, but showed an apparent decrease after hydroxylation (Figure S5). The most characteristic vibration of PS is the out-of-plane mode of the mono-substituted aromatic group located at ~700 cm^−1^, which is not overlaying with other signals, and so can be used as internal reference [63].

Because the *T_g_* is exceeded, at *T* > 110 °C the baseline of higher wavenumbers is shifted partially to lower absorption values. This effect is also influenced by minimal differences in sample preparation, and was not always observed. This effect is not related to changes of the absorption coefficient due to temperature induced change of chemical structure.

#### Temperature Dependent FTIR Spectroscopy of I_1.5_S_96.1_I_2.4_^82^-SA and I_5_S_90_I_5_^62^-DETA

**Figure 12:**
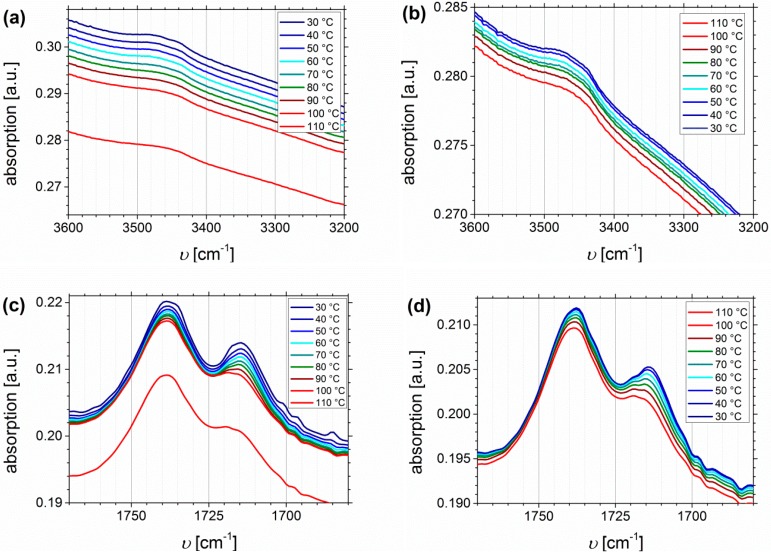

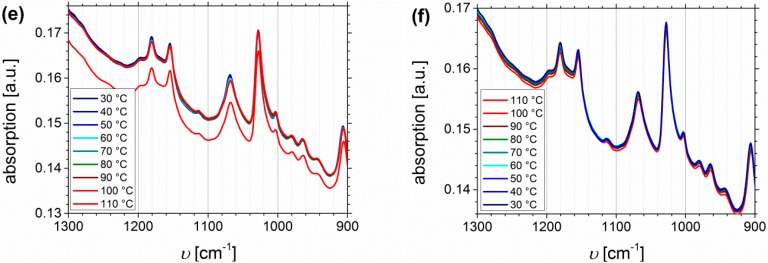
Temperature dependent FTIR spectra of I_1.5_S_96.1_I_2.4_^82^-SA (*D_f_* = 33%). Temperature range was 30 to 110 to 30 °C with 10 °C steps, and a 15 min isothermal hold at each temperature ((**a**,**c**,**e**): Heating curves; (**b**,**d**,**f**): Cooling curves).

The thermo-reversible behavior will be investigated up to 110 °C, including the heating and cooling process. The main focus will be on SA- and DETA-functionalized block copolymers, due to the stronger ability to form hydrogen bonds, and due to related future publications on dynamic mechanical analysis. Both presented block copolymers show quite similar *D_f_* values (32% and 33%, respectively), but it has to be considered that the absolute number of functional groups is varying due to different PI contents in the original polymers. Figure 12 shows the temperature dependent FTIR spectra of I_1.5_S_96.1_I_2.4_^82^-SA (*D_f_* = 33%). Plotted are the regions of O-H stretching vibrations (Figure 12a,b), the carbonyl region (Figure 12c,d), and the fingerprint region at 1300–900 cm^−1^, characteristic of C-O stretching vibrations (Figure 12e,f).

From the O-H stretching vibration region in the spectra of I_1.5_S_96.1_I_2.4_^82^-SA, no significant information can be deduced. In the carbonyl region (Figure 12c,d) two main signals occur. With increasing temperature the decrease, and the slight high-frequency shift of the signal at 1715 cm^−1^ is stronger than that of the signal at 1738 cm^−1^. This indicates that 1715 cm^−1^ is assigned to the carboxyl group, 1738 cm^−1^ can be related to the ester group, and that both carbonyl groups are not equivalent according to hydrogen bond formation, for example, because the ester bond is sterically hindered. In literature [64,65,66], the infrared absorbance at 1700 cm^−1^ is assigned to the dimer (complexed species), and 1750 cm^−1^ to the monomer (free species) carbonyl stretch, which is consistent with the observations from this work. Therefore, it is concluded that the free species of the carboxyl-related signal is overlapping with the ester-related signal, and thus, cannot be detected. In general, hydrogen bonded carboxylic groups can form different arrangements like dimers, trimers, or catemers [67,68]. Compared, for example, to the work of Wittenberg et al. [69], where for statistically functionalized triblock copolymers several complexes were identified also at wavenumbers lower than 1700 cm^−1^, in this work only dimeric complexes seem to be detected. This as well might be explained by the steric properties, and hence, the arrangement of the motif. More specific conclusions about the formation of different structures cannot be made. In the fingerprint region, no significant changes can be observed.

**Figure 13:**
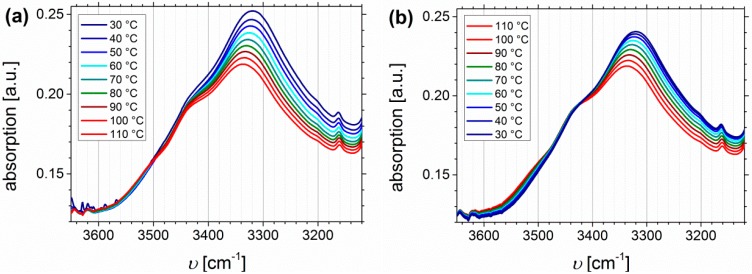

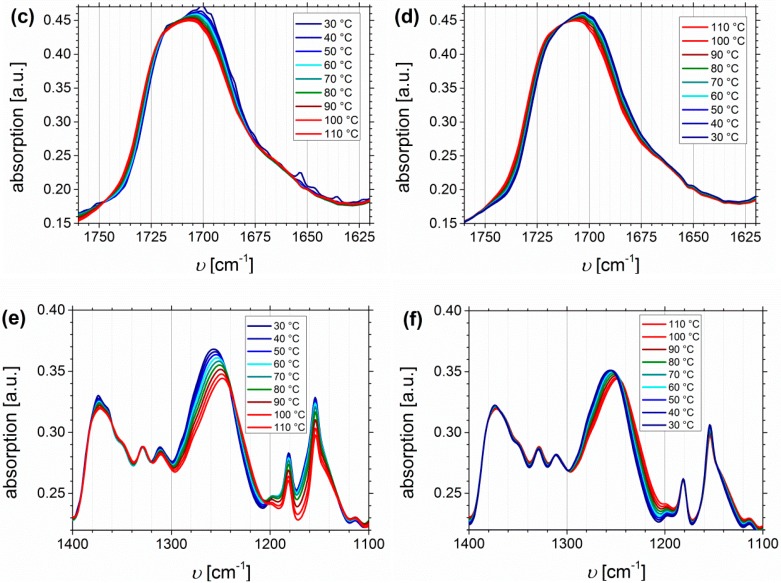
Temperature dependent FTIR spectra of I_5_S_90_I_5_^62^-DETA (*D_f_* = 32%). Temperature range was 30 to 110 to 30 °C with 10 °C steps, and a 15 min isothermal hold at each temperature ((**a**,**c,e**): Heating curves; (**b**,**d**,**f**): Cooling curves).

Figure 13 shows the temperature dependent FTIR spectra of I_5_S_90_I_5_^62^-DETA (*D_f_* = 32%), again divided into the region of N-H stretching vibrations (Figure 13a,b), the carbonyl region (Figure 13c,d), and the fingerprint region between 1400 and 1100 cm^−1^ related to C-N and C-O stretching (Figure 13e,f).

In contrast to the O-H stretching vibration of carboxylated ISI, much better thermo-responsiveness is detectable for the N-H stretching vibration band, which could be explained by the larger number of functional groups (due to different PI contents of the original polymer). With increasing temperature the band of the associated species at 3340 cm^−1^ decreases, whereas the region above 3500 cm^−1^, corresponding to the free species, slightly increases. This observation is in good agreement with literature [59,70,71]. During the cooling process, the opposite behavior can be observed, which verifies the reversible characteristic of this polymer. For polyurethanes and triazolindione functionalized polymers, which are comparable to the investigated motif, the hydrogen-bonded and non-hydrogen-bonded carbonyl bands are located at 1660–1703 and 1723–1740 cm^−1^, respectively [14,59,71,72]. These bands can be clearly distinguished in the presented system, and also show thermo-reversible behavior, but less than the N-H stretching vibrations. Again, the sterical hindrance of the carbonyl group inside the motif can be the explanation. In contrast to ISI-SA, where too many bands are overlapping, for the DETA-functionalized polymers two isosbestic points are observable at 1714 cm^−1^, and in the fingerprint region at 1244 cm^−1^.

Due to the *T_g_* of the main polymer component PS at around 100 °C, it was expected that the polymer chains are frozen at *T* < *T_g_*, and so no or a lower amount of hydrogen bonds can be formed. With decreasing amount of functionalized PI, and thus less number of hydrogen bonding donor and acceptor groups which could meet, less temperature dependent behavior was anticipated. Even if for particular samples this has been observed, for most samples hydrogen bonds were thermo-reversible formed. Usually, the polymer films showed directly after solution casting onto the KBr pellets the highest content of hydrogen bonds. It is presumed that the microphase separation enhances the probability of hydrogen bond formation, as the donor and acceptor groups show a lower distance. Perhaps a further increase of the PS block above a certain content would impair the formation of hydrogen bonds. 

## 4. Conclusions

The synthesis of this novel approach of end-block functionalized ISI triblock copolymers was accomplished, and different hydrogen bond forming groups—containing hydroxylic, carboxylic or amine moieties—were successfully introduced. It was observed that the degree of hydroxylation for low PI contents (less than 5 wt %) did not reach 100%, presumable due to the miscibility of PS and PI blocks, and the resulting sterical hindrance. Further modifications with succinic anhydride (SA) or diethylenetriamine (DETA) showed much better conversions than, for example, 2,6-diaminopyridine (DAP). FTIR spectroscopy studies of ISI-SA and ISI-DETA indicated the thermo-reversible behavior up to 110 °C. The temperature dependent dynamic mechanical properties of these thermo-reversible networks will be the topic of another publication.

## Figures and Tables

**Table 1:** Investigated di- and triblock copolymers and the dispersity indices of precursor *Đ*_Pre_ as well as *Đ*_poly_ of the resulting SI or ISI block copolymers. All *Đ* were determined from SEC measurements. PI-Precursors were not measured due to too low molecular weight. The degree of polymerization *P*_n_ is given for the polystyrene (S) and polyisoprene (I) monomer units.

Polymer	*Đ*_S-Pre_	*Đ*_SI_	*P*_n_ (S/I)	Polymer	*Đ*_IS-Pre_	*Đ*_ISI_	*P*_n_ (I/S/I)
S_91_I_9_^67^	1.1	1.1	585/89	I_5_S_90_I_5_^62^	1.2	1.3	46/536/46
S_85_I_15_^51^	1.4	1.4	416/112	I_3_S_94_I_3_^117^	1.2	1.2	52/1056/52
S_41_I_59_^31^	1.1	1.1	123/273	I_1.5_S_96.1_I_2.4_^82^	1.2	1.2	18/757/29
S_51_I_49_^5^	1.3	1.1	24/35	I_0.6_S_98.8_I_0.6_^98^	1.2	1.2	9/935/9
				I_0.7_S_98.5_I_0.8_^149^	1.2	1.4	10/932/11

**Table 2:** Measured and calculated data of different functionalized and unfunctionalized ISI and SI. Listed are the polymeric precursor, the introduced functional group FG_int_, the degree of functionalization *D_f_*, the polydispersity index *Đ*, and the onset of glass transition temperature *T_g_*. Modified samples are listed directly after their OH- or CDI-functionalized precursor.

Polymer	FG_int_	*Đ*	*D_f_* [%]	*T_g_* [°C] *^a^*
I_5_S_90_I_5_^62^	-	1.3	-	86.0
I_5_S_90_I_5_^62^	OH	1.2	58	93.2
I_5_S_90_I_5_^62^	OH	1.3	70	*^c^*
I_5_S_90_I_5_^62^	OH	1.3	94	101.5
I_5_S_90_I_5_^62^	DETA	*^b^*	32	97.8
I_5_S_90_I_5_^62^	TETA	1.4	21	94.8
I_5_S_90_I_5_^62^	DAP/CDI	1.3	10/7	100.7
I_5_S_90_I_5_^62^	SA	2.0	45	97.3
I_1.5_S_96.1_I_2.4_^82^	–	1.2	–	95.0
I_1.5_S_96.1_I_2.4_^82^	OH	1.2	75	97.9
I_1.5_S_96.1_I_2.4_^82^	DETA	1.3	48	98.9
I_1.5_S_96.1_I_2.4_^82^	SA	1.2	33	100
I_0.6_S_98.8_I_0.6_^98^	–	1.2	–	101.8
I_0.6_S_98.8_I_0.6_^98^	OH	1.2	43	102.5
I_0.6_S_98.8_I_0.6_^98^	DETA	1.3	13	102.0
I_0.6_S_98.8_I_0.6_^98^	SA	1.2	2	104.0
I_3_S_94_I_3_^117^	–	1.2	–	83.7
I_3_S_94_I_3_^117^	OH	1.5	50	101.1
I_3_S_94_I_3_^117^	DETA	2.3	20	98.2
I_3_S_94_I_3_^117^	SA	1.7	30	99.8
I_0.7_S_98.5_I_0.8_^149^	–	1.2	–	99.6
I_0.7_S_98.5_I_0.8_^149^	OH	1.2	83	103.0
I_0.7_S_98.5_I_0.8_^149^	DETA	1.5	29	102.0
I_0.7_S_98.5_I_0.8_^149^	SA	1.2	14	102.0
S_85_I_15_^51^	–	1.4	–	*^c^*
S_85_I_15_^51^	OH	1.5	27	*^c^*
S_85_I_15_^51^	CDI	1.5	11	*^c^*
S_85_I_15_^51^	DETA	2.4	12	*^c^*
S_85_I_15_^51^	OH	1.8	100	*^c^*
S_85_I_15_^51^	CDI	*^c^*	18	*^c^*
S_85_I_15_^51^	DAP	1.6	4	*^c^*
S_91_I_9_^67^	–	–	–	98.2
S_91_I_9_^67^	OH	1.2	30	99.1
S_91_I_9_^67^	SA	1.4	11	100.8
S_91_I_9_^67^	OH	1.1	92	97.8
S_91_I_9_^67^	SA	1.5	45	99.0

*^a^* Onset; *^b^* not analyzed (due to insolubility); *^c^* not measured.

## Supplementary Materials

The following are available online at http://www.mdpi.com/1996-1944/11/9/1608/s1, Figure S1: ^1^H NMR spectra of PI-Precursor (red), PI-*b*-PS-Precursor (green) and I_5_S_90_I_5_^62^ (blue) in CDCl_3_. Spectra were normalized to aromatic protons of polystyrene (6.2–7.2 ppm, 5H), Figure S2: ^1^H NMR spectra of S_91_I_9_^67^ (black), and after hydroxylation with different degree of modification in CDCl_3_. Hydroxylation of 3,4-PI (signal at 4.45–4.65 ppm) is favoured. Spectra were normalized to aromatic protons of polystyrene (6.2–7.2 ppm, 5H), Figure S3: ^1^H NMR spectra of unfunctionalized (black), hydroxylated (blue) and with SA carboxylated (red) I_5_S_90_I_5_^62^ in CDCl_3_. Spectra were normalized to aromatic protons of PS (6.2–7.2 ppm, 5H), Figure S4: (a) ^1^H NMR spectra of S_85_I_15_^51^ (black), after hydroxylation (blue), and after reaction with CDI (orange) and DETA (green) in CDCl_3_. (b) ^1^H NMR spectra of CDI-functionalized S_85_I_15_^51^ (top), and after addition of DAP with reaction times of 7 h, 3 d and 4 d (from top to the bottom) in CDCl_3_. ^1^H NMR spectra were normalized to aromatic protons of PS (6.2–7.2 ppm, 5H)., Figure S5: ATR-FTIR spectra of S_91_I_9_^67^ before (black) and after (blue) hydroxylation (*D_f_* = 30%). Characteristic decrease of PI related bands can be observed, for example, at 1645 and 890 cm^−1^. Both bands can be assigned to the main 3,4-addition product of PI. (a) Complete spectra and (b) enlarged area of (a) at 1800–800 cm^-1^, Figure S6: Full range temperature dependent FTIR spectra of I_1.5_S_96.1_I_2.4_^82^-SA (*D_f_* = 33%). Temperature range was 30 to 110 (a) and to 30 °C (b) with 10 °C steps, and a 15 min isothermal hold at each, Figure S5: Full range temperature dependent FTIR spectra of I5S90I562-DETA (*D_f_* = 32%). Temperature range was 30 to 110 (a) to 30 °C (b) with 10 °C steps, and a 15 min isothermal hold at each temperature.
