# Supplementary material for: Supramolecular Networks from Block Copolymers Based on Styrene and Isoprene Using Hydrogen Bonding Motifs—Part 1: Synthesis and Characterization
Source: Materials (Basel). 2018 Sep 4;11(9):1608. doi: 10.3390/ma11091608 (PMC6164727; doi:10.3390/ma11091608)
Supplement: Supplementary file 1 [file materials-11-01608-s001.pdf]

Supplementary Information

Supramolecular Networks from Block Copolymers Based on Styrene and Isoprene Using Hydrogen Bonding Motifs – Part 1: Synthesis and Characterization

Elaine Rahmstorf [1], Volker Abetz [1,2,*]

[1] Institute of Physical Chemistry, University of Hamburg, Grindelallee 117, 20146 Hamburg, Germany; elaine.rahmstorf@chemie.uni-hamburg.de

[2] Institute of Polymer Research, Helmholtz-Zentrum Geesthacht, Max-Planck-Straße 1, 21502 Geesthacht, Germany; volker.abetz@hzg.de

* Correspondence: volker.abetz@hzg.de; Tel.: +49 40 42838-3460

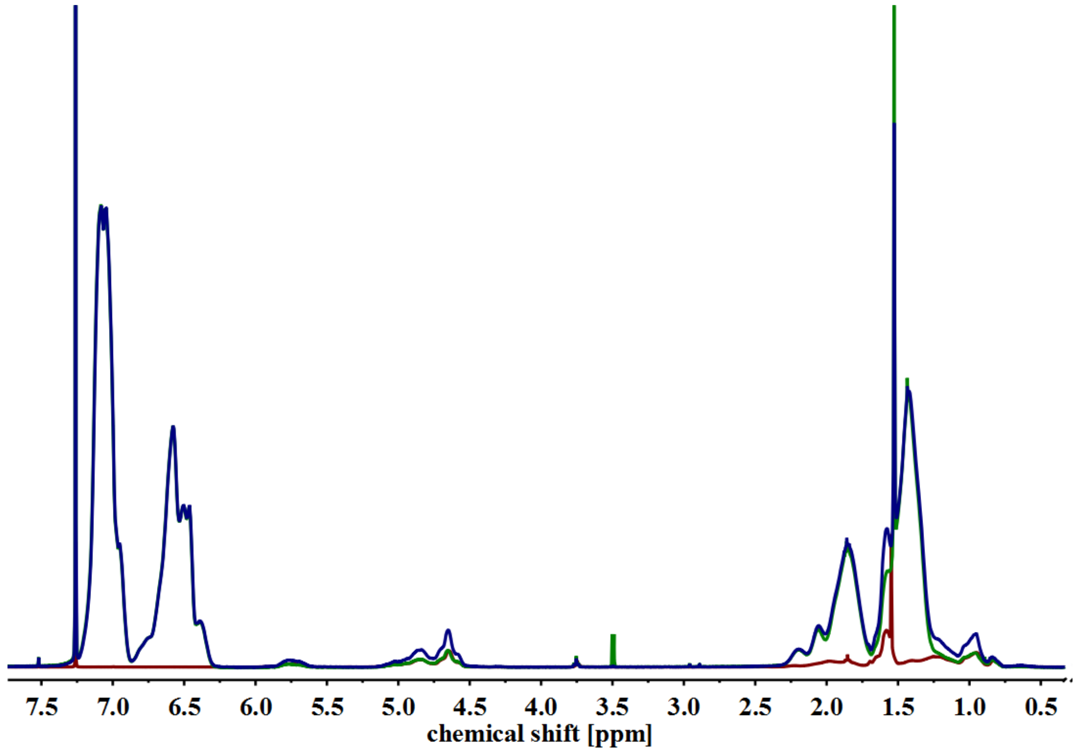

Figure S1: ^{1}H NMR spectra of PI-Precursor (red), PI-*b*-PS-Precursor (green) and $I_5S_{90}I_5^{62}$ (blue) in $CDCl_3$. Spectra of PI-Precursor and PI-*b*-PS-Precursor were normalized to PI signals. Spectra of PI-*b*-PS-Precursor and $I_5S_{90}I_5^{62}$ were normalized to aromatic protons of polystyrene (6.2–7.2 ppm, 5H).

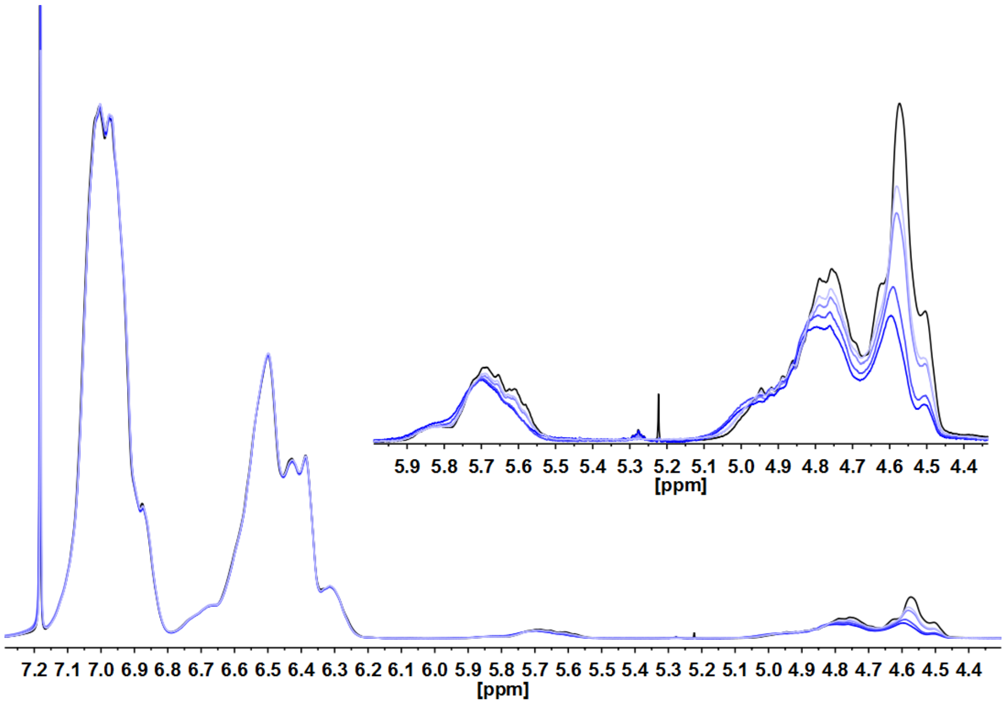

Figure S2: ^{1}H NMR spectra of $S_{91}I_{9}^{67}$ (black), and after hydroxylation with different degree of modification (0%, 13%, 18%, 29%, and 35%) in $CDCl_3$. Hydroxylation of 3,4-PI (signal at 4.45–4.65 ppm) is favoured. Spectra were normalized to aromatic protons of polystyrene (6.2–7.2 ppm, 5H).

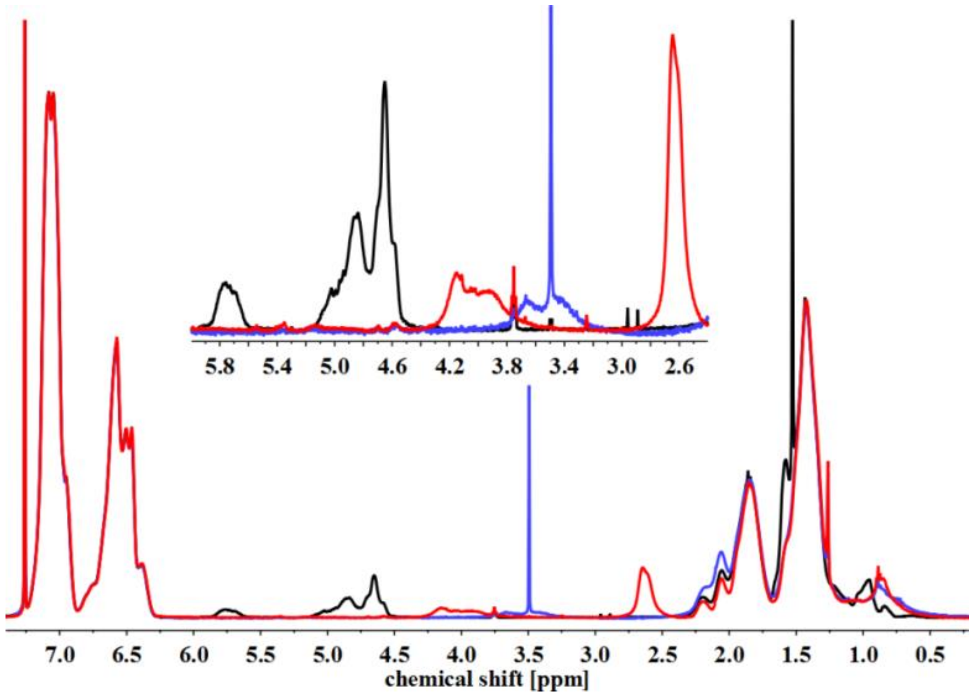

Figure S3: ^{1}H NMR spectra of unfunctionalized (black), hydroxylated (blue) and with succinic anhydride carboxylated (red) $I_5S_{90}I_5^{62}$ in $CDCl_3$. Spectra were normalized to aromatic protons of PS (6.2–7.2 ppm, 5H).

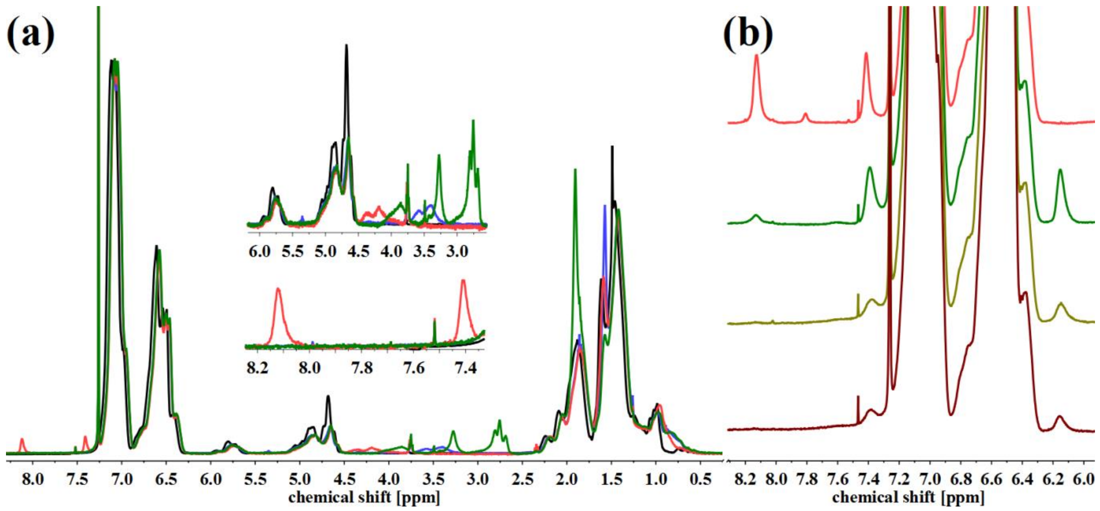

Figure S4: (**a**) ^{1}H NMR spectra of $S_{85}I_{15}{}^{51}$ (black), after hydroxylation (blue), and after reaction with CDI (orange) and DETA (green) in $CDCl_3$. (**b**) ^{1}H NMR spectra of CDI-functionalized $S_{85}I_{15}{}^{51}$ (top), and after addition of DAP with reaction times of 7 h, 3 d and 4 d (from top to the bottom) in $CDCl_3$. ^{1}H NMR spectra were normalized to aromatic protons of PS (6.2–7.2 ppm, 5H).

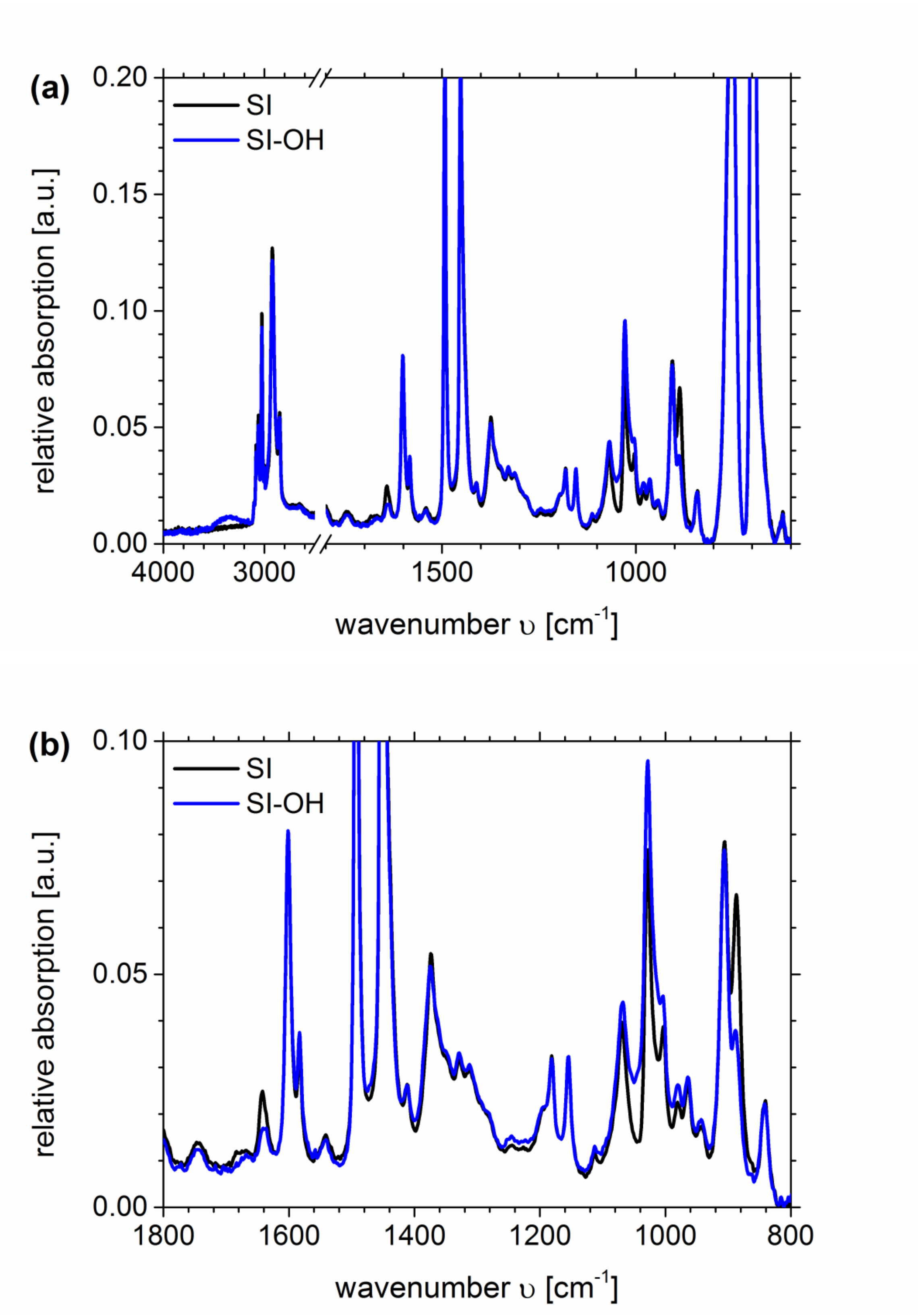

Figure S5: ATR-FTIR spectra of $S_{91}I_9^{67}$ before (black) and after (blue) hydroxylation (D_f = 30%). Characteristic decrease of PI related bands can be observed, for example, at 1645 and 890 cm^{-1}. Both bands can be assigned to the main 3,4-addition product of PI. (**a**) Complete spectra and (**b**) enlarged area of (a) at 1800–800 cm^{-1}.

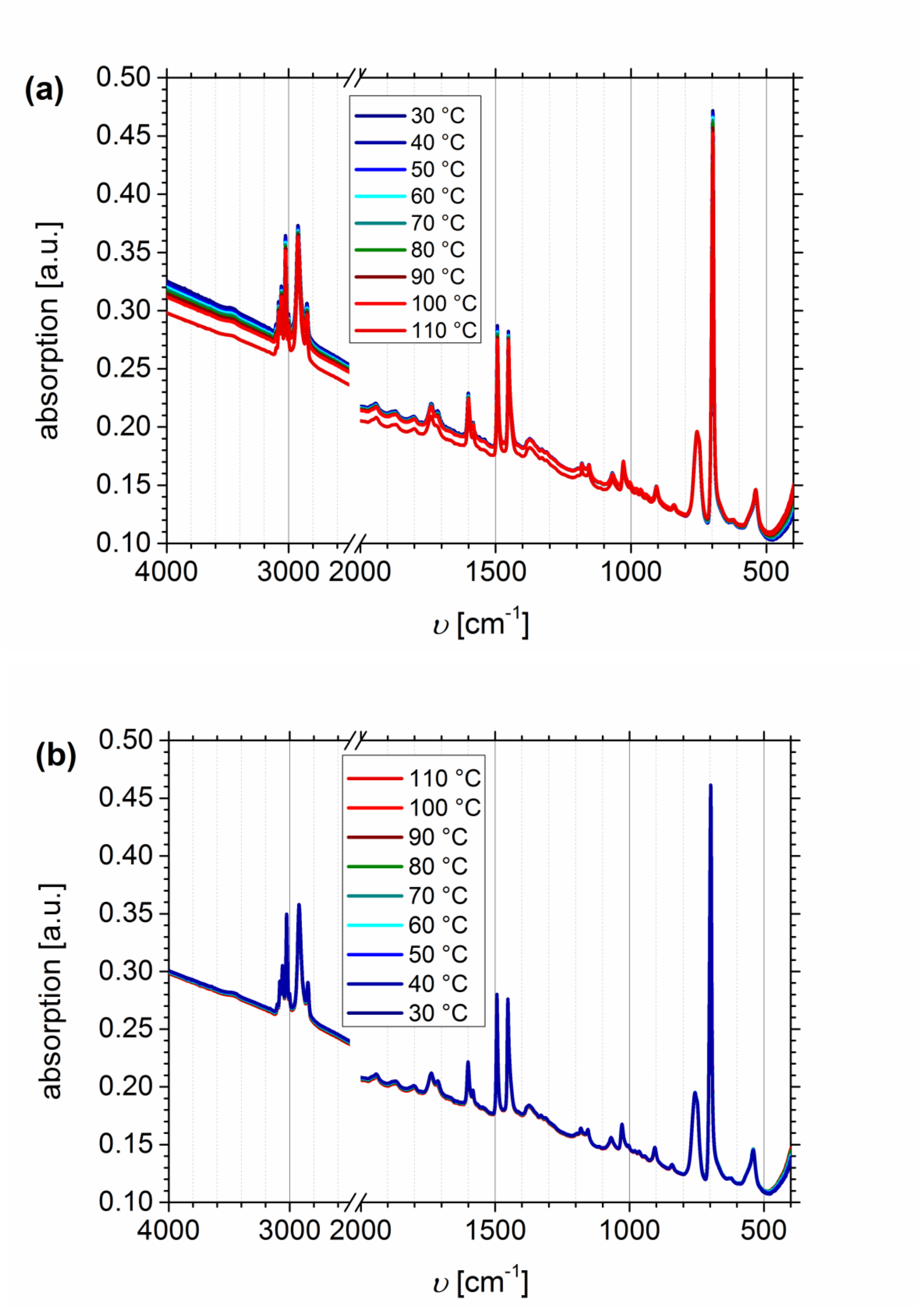

Figure S6: Full range temperature dependent FTIR spectra of $I_{1.5}S_{96.1}I_{2.4}{}^{82}$-SA ($D_f$ = 33%). Temperature range was 30 to 110 (**a**) and to 30 °C (**b**) with 10 °C steps, and a 15 min isothermal hold at each temperature.

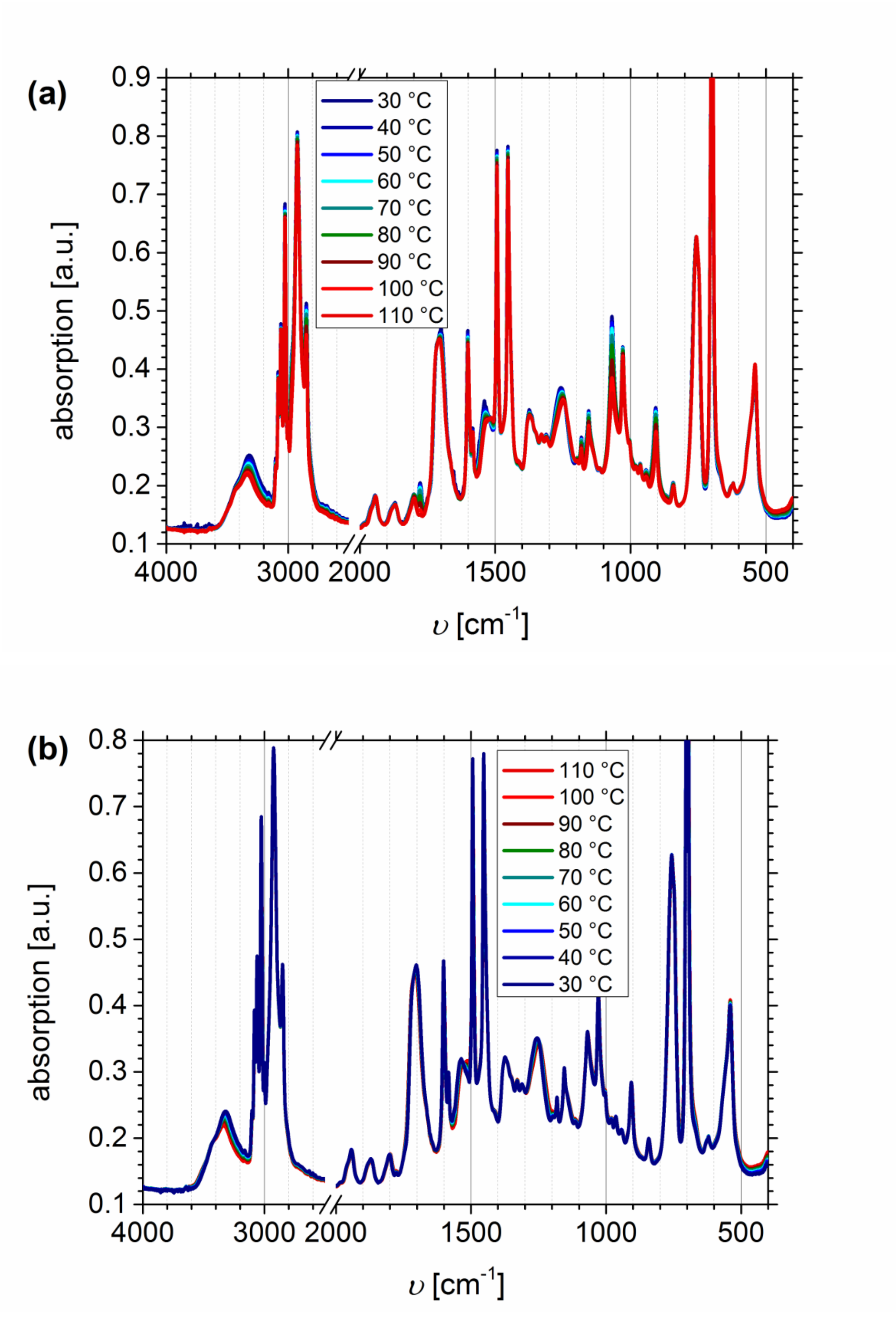

Figure S7: Full range temperature dependent FTIR spectra of $I_5S_{90}I_5^{62}$-DETA (D_f = 32%). Temperature range was 30 to 110 (**a**) and to 30 °C (**b**) with 10 °C steps, and a 15 min isothermal hold at each temperature.
